# A four-year assessment of the characteristics of Rwandan FDA drug recalls

**DOI:** 10.1186/s12889-024-19245-8

**Published:** 2024-07-04

**Authors:** Marcel Bahizi, Eric Nyirimigabo, Lazare Ntirenganya, Martine Ishimwe Umuhoza, Vedaste Habyalimana, Gerard Bikorimana, Joyeuse Ukwishaka

**Affiliations:** 1 Rwanda Food and Drugs Authority, Kigali, Rwanda; 2 University of Rwanda, Kigali, Rwanda; 3 Kigali Independent University, Kigali, Rwanda; 4 Biomedical Center/ Maternal Child and Community Health Division, Kigali, Rwanda

**Keywords:** Drug recall, Rwanda FDA, Quality issues, Class of recall

## Abstract

**Background:**

A drug recall is an act of removing products from the market and/or returning them to the manufacturer for disposal or correction when they violate safety laws. Action can be initiated by the manufacturing company or by the order of a regulatory body. This study aimed to assess the characteristics of Rwanda FDA drug recall and determine the association between classes of recall and recall characteristics.

**Methodology:**

This was a retrospective descriptive cross-sectional study. Data about recalled drugs were collected from the official website of the Rwanda FDA in the section assigned to “Safety alerts”. The search included data reported between February 2019 and February 2023 covering four years. Data cleaning was conducted in Microsoft Excel to address missing data and inconsistencies, followed by importation into STATA/SE software version 17.0 for further cleaning and subsequent analysis. Descriptive statistics were computed for independent variables. Categorical variables were described in terms of counts and relative frequencies. Bivariate analyses used Pearson’s chi-square test to illustrate the associations between categorical independent variables and recall classes.

**Results:**

The study revealed that a large proportion (33.0%) of the recalled products belonged to Class I. Antibiotics constituted 35.8% of the recalled products, with contamination emerging as a leading cause and responsible for 26.4% of the recalls. India was the leading manufacturing country for the recalled products (29.2%), followed by France (17.9%), China (17.0%), Kenya (13.2%), and Russia (6.6%). An association was found between the class of recall and several recall characteristics, including the year of recall, drug category, safety issues, reporter, and manufacturing country.

**Conclusion:**

This study provides a comprehensive overview of the characteristics of drug recalls in Rwanda. The insights gained contribute to a nuanced understanding of recall dynamics and provide evidence-based strategies to enhance drug quality, safety, efficacy, regulatory compliance, and patient welfare.

**Supplementary Information:**

The online version contains supplementary material available at 10.1186/s12889-024-19245-8.

## Background

A drug recall is an act of removing from the market and/or returning to the manufacturer for disposal or correction the products that violate safety laws [1, 2]. The action can be initiated by the manufacturing company or by the order of a regulatory body [1–3]. Drug recall imposes financial and economic negative consequences for the manufacturing company in addition to the long-term market crisis if the recall decreases consumers’ trust in the concerned company [4].

Drug recall is a crucial measure for ensuring the quality of the products available on the market while protecting the public [5]. Product recall can be due to adverse effects, mislabelling or poor packaging, defective product, incorrect potency, or contamination [2, 4, 6, 7].

There are three categories of drug recalls considering their severity: Class I recall: if the use of the product will cause death or dangerous harmful health consequences; Class II recall: if the use of the product will cause temporary or reversible harmful health consequences; and Class III recall: if the use of the product is not likely to cause harmful health consequences and may be linked to the container design problem [2–5, 8].

The Rwanda Food and Drug Authority (FDA) was established by the law N^o^ 003/2018 of 09/02/2018. Its mandate is to protect public health by regulating human and veterinary medicines, vaccines, medical devices, biological products, processed foods, household chemicals, poisons, medicated cosmetics, and tobacco products [9].

Since its establishment in 2018, the Rwanda FDA has contributed to managing, assuring, and controlling the quality of the products under its regulatory mandate [10]. During these four years, the Rwanda FDA has recalled different products from the market, either from the initiative of the Rwanda FDA or the companies [10, 11].

Rwanda FDA communicates product safety alerts to the public using press releases, which are made available on Rwanda FDA website. The recall reports contain the reasons for the recall and advice to healthcare providers and consumers.

However, this information is available for individual product recalls and no comprehensive analysis has been conducted to evaluate the specifics of these recalls or the distribution of recalls among manufacturing companies. Conducting such an analysis is essential for improving public awareness.

This study aimed to assess the characteristics of Rwanda FDA recall products and determine the association between the classes of recall and the recall characteristics. The study’s objectives were to a) Identify the types and categories of products recalled by the Rwanda FDA, b) Determine the frequency and trends of product recalls over a four-year period, c) Analyse the distribution of recalls across different product categories, d) Identify the primary reasons for product recalls, e) Evaluate the association between recall class and product categories, and f) Provide recommendations based on the findings to improve consumer safety and regulatory processes.

## Methods

### Study design

This was a retrospective descriptive cross-sectional study. Data related to drug recalls were collected from the official website of the Rwanda Food and Drug Authority (FDA) in the section assigned to “Safety alerts”. The data collection period encompassed reports from February 2019 to February 2023, spanning four years.

### Data collection

All available reports and press releases on product recalls, both downloadable and non-downloadable, were included in this study. Reports or press releases with missing data regarding key variables of interest, namely the class of recall, reason for recall, product category, and manufacturing company, were excluded from the analysis.

### Data variables

The collected data contained comprehensive information about the recalled products, including details such as manufacturing company, product category, active ingredient (where applicable), affected batch number, reason for recall, date of recall, reporter, supplier, and actions taken by the Rwanda FDA. To avoid duplicates, each recall was uniquely identified and recorded.

### Data cleaning

Data cleaning procedures were carried out using Microsoft Excel. This involved addressing issues related to missing data and inconsistencies. Subsequently, the data were imported into STATA/SE software version 17.0 for further cleaning, which included recording and generating new variables of interest. The primary variables of interest included the class of recall (Class I, Class II and Class II), the reason for the recall, and the manufacturing country, the product group, which were categorized according to the WHO’s Anatomical Therapeutic Chemical (ATC) coding system [12]. The World Health Organization (WHO), through its Collaboration Center for Drug Statistics Methodology (WHOCC), oversees the ATC Classification System [12]. This system categorizes the active ingredients of drugs on the basis of their therapeutic, pharmacological, and chemical properties, as well as the organ or system they target [12]. Additionally, WHO’s AWaRe classification categorizes antibiotics into three groups: Access, Watch, and Reserve, reflecting their importance and the necessity for stewardship. This classification system is designed to guide efforts to promote appropriate use of antibiotics and address the growing concern of antimicrobial resistance [13].

### Statistical analysis

Descriptive statistics were applied to analyse independent variables, with categorical variables presented using counts and relative frequencies. Bivariate analyses were conducted using Pearson’s chi-square test to explore associations between categorical independent variables and the class of recall. Statistical significance was set at a p-value of < 0.05.

## Results

### Characteristics of drug recalls

During the four-year assessment, a diverse array of characteristics emerged among the recalled products (Table S1). Analysis using the ATC coding system indicated that a significant proportion of recalled products belonged to group A (33.1%), followed by group J (27.4%), and group C (8.5%). Notably, within the drug category analysis, antibiotics constituted 35.8% of the recalled products, spasmolytics accounted for 16.9%, disinfectants and antiseptics 11.3%, and antihypertensive 7.5% of the recalls. The majority (71.8%) of recalled antibiotics belonged to the “Access” group of AWaRe, WHO’s classification (refer to Tables 1, 2 and 3).

**Table 1:** Characteristics of recalls: drug category vs ATC classification

**Recall characteristics**			**Total**
**Drug category**	**ATC Classification**	**n (%)**	**N (%)**
Antibiotic	**J** (Anti-infective for Systemic use)	29 (27.3)	38 (35.8)
	**A** (Anti-infective for intestinal Infections)	7 (6.6)	
	**G** (Anti-infective for Genito-urinary system)	1 (0.9)	
	**D** (Dermatological)	1 (0.9)	
Anti-inflammatory/ analgesics/antipyretics	**N** (analgesics / antipyretics acting on nervous system)	4 (3.8)	5 (4.7)
	**H** (Systemic steroid)	1 (0.9)	
Disinfectants / Antiseptics	**D** (Topical antiseptics)	9 (8.5)	12 (11.3)
	**V** (Technical, surface disinfectants)	3 (2.8)	
Spasmolytic	**A** (Oral spasmolytic)	18 (16.9)	18 (16.9)
Antiparasitic	**P** (Anti-parasitic products)	4 (3.8)	4 (3.8)
Antihypertensive	**C** (Cardiovascular system drugs)	8 (7.5)	8 (7.5)
Vitamins	**A** (Oral multivitamins)	5 (4.7)	6 (5.7)
	**B** (Vitamin for Blood clotting)	1 (0.9)	
Diagnostic agent	**V** (Chemicals and reagents for analysis)	5 (4.7)	5 (4.7)
Others	-	10 (9.4)	10 (9.4)

**Table 2:** Characteristics of drug recalls: antibiotics AWaRe classification vs supplier

**Characteristics of recalls**			**Total**
**Antibiotics AWaRe category (** ***N*** ** = 38)**	**Supplier**	**n (%)**	**n (%)**
Access	Non-governmental	16 (42.1)	27 (71.0)
	Governmental	11 (28.9)	
Watch	Non-governmental	4 (10.5)	4 (10.5)
Unclassified*	Non-governmental	6 (2.7)	7 (18.4)
	Others	1 (15.8)	

^*^ **Unclassified antibiotics**: They are either antibiotics that have not yet been categorized in the AWaRe classification or fixed-dose combinations of multiple broad-spectrum antibiotics [37]. The use of fixed-dose combinations is not recommended by the WHO because of a lack of evidence, and they may contribute to increased antimicrobial resistance [37–39]. In this study, the unclassified category included aminosidine antibiotics that are not listed on either the Essential Medicines List (EML) and the AWaRe classification list

**Table 3:** Characteristics of drug recalls: drug category vs supplier

**Recall characteristics**			**Total**
**Drug category**	**Supplier**	**n (%)**	**N (%)**
Antibiotic	Non-governmental	26 (24.5)	38 (35.8)
	Governmental	7 (6.6)	
	Others	1 (0.9)	
Anti-inflammatory/ analgesics/antipyretics	Non-governmental	3 (2.8)	5 (4.7)
	Others	2 (1.8)	
Disinfectants / Antiseptics	Non-governmental	4 (3.8)	12 (11.3)
	Others	8 (7.5)	
Spasmolytic	Non-governmental	18 (16.9)	18 (16.9)
Antiparasitic	Non-governmental	2 (1.8)	4 (3.8)
	Governmental	1 (0.9)	
	Others	1 (0.9)	
Antihypertensive	Non-governmental	2 (1.8)	8 (7.5)
	Others	6 (5.7)	
Vitamins	Non-governmental	1 (0.9)	6 (5.7)
	others	5 (4.7)	
Diagnostic agent	Non-governmental	5 (4.7)	5 (4.7)
Others	-	10 (9.4)	10 (9.4)

The reasons for product recalls encompass a spectrum of safety concerns. Notably, contamination emerged as a leading cause, accounting for 26.4% of all recalled products. The apprehension surrounding suspected poor quality prompted the recall of 18.9% of products. Mislabeling constituted a distinct factor driving recalls and was responsible for 8.5% of the total cases. Additionally, an array of specific circumstances contributed to the remaining recalls. Instances of self-opening capsules, color changes, and clumping each accounted for 7.5% of recalls. Furthermore, precipitation accounted for 6.6% of recalls (Table 4).

**Table 4:** Characteristics of drug recalls: quality issue vs reporter

**Recall characteristics**			
**Quality issue**	**Reporter**	**n (%)**	**N (%)**
Contamination	Private pharmacies	20 (18.9)	28 (26.4)
	Rwanda Medical Supply Ltd	7 (6.6)	
	Others	1 (0.9)	
Suspected poor quality	Private pharmacies	7 (6.6)	20 (18.9)
	Rwanda Medical Supply Ltd	4 (3.8)	
	Rwanda FDA	8 (7.5)	
	Others	1 (0.9)	
Self-opening capsules	Private pharmacies	3 (2.8)	8 (7.5)
	Public pharmacies	5 (4.7)	
Not labelled	Rwanda FDA	3 (2.8)	9 (8.5)
	Public pharmacies	6 (5.7)	
Precipitation	Rwanda FDA	7 (6.6)	7 (6.6)
Color change	Private pharmacies	2 (1.9)	8 (7.5)
	Rwanda FDA	2 (1.9)	
	Others	4 (3.7)	
Clumping	Rwanda Medical Supply Ltd	8 (6.6)	8 (6.6)
Others	-	18 (17.0)	18 (17.0)

In terms of the class of recall, the distribution was as follows: class I recalls accounted for 33.0%, class II recalls represented the majority at 58.5%, and class III recalls at 8.5%. Supplier categorization revealed that non-governmental entities supplied most of the recalled products (63.2%), and governmental entities supplied 13.2%.

The international perspective showcased India as the leading manufacturing country for the recalled products (29.2%), followed by France (17.9%), China (17%), Kenya (13.2%), and Russia (6.6%) (Table 5).

**Table 5:** Characteristics of drug recalls: manufacturing country vs safety issues

**Recall characteristics**			**Total**
**Manufacturing country**	**Safety issues**	**n (%)**	**N (%)**
India	Contamination	6 (5.7)	31 (29.2)
	Suspected poor quality	8 (7.5)	
	Color change	1 (0.9)	
	Clumping	8 (7.5)	
	Others	8 (7.5)	
France	Contamination	19 (17.9)	19 (17.9)
China	Self-opening capsule	8 (7.5)	18 (17.0)
	Not labelled	6 (5.7)	
	Others	4 (3.8)	
Kenya	Contamination	1 (0.9)	14 (13.2)
	Suspected poor quality	11 (10.3)	
	Color change	7 (6.6)	
	Others	5 (4.7)	
Russia	Precipitation	7 (6.6)	7 (6.6)
Others	-	17 (16.0)	17 (16.0)

The accountability for reporting recalls exhibited a diverse landscape. Rwanda FDA reported 23.6% of the recalls, while Rwanda Medical Supply Ltd reported 19.8%. Private pharmacies and firms contributed significantly, reporting 36.8% of recalls, and public or government pharmacies reported 10.4% of the recalls. Markedly, the products under recall were predominantly at the retail level (95.3%), while only a minor percentage pertained to the consumer level (4.7%) (Table 6).

**Table 6:** Characteristics of drug recalls: recall level vs safety issues

**Recall characteristics**			**Total**
**Recall level**	**Safety issues**	**n (%)**	**N (%)**
Retail	Contamination	28 (26.4)	101 (95.3)
	Suspected poor quality	15 (14.1)	
	Self-opening capsule	8 (7.5)	
	Not labelled	9 (8.5)	
	Precipitation	7 (6.6)	
	Color change	8 (7.5)	
	Clumping	8 (7.5)	
	Others	18 (17.0)	
Consumer	Suspected poor quality	5 (4.7)	5 (4.7)

### Recall trends over the four years

**Fig. 1:**
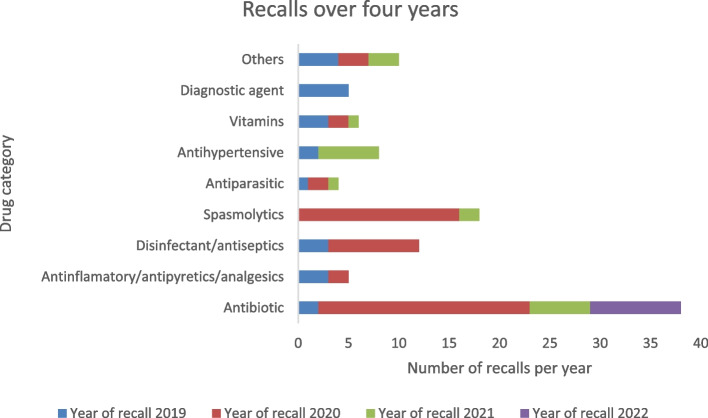
Recalls per year

The data revealed distinct recall trends over the course of four years. In 2019, approximately 21.7% of products were recalled from the market. Subsequently, in 2020, a more substantial percentage of products (51.9%) underwent recalls. 2021 witnessed a regression in recall incidents, with 17.9% of products being subject to recalls. Finally, in 2022, the recall frequency further decreased, with 8.5% of products being recalled. In the year 2020, antibiotics and spasmolytics were the most recalled products (Fig. 1).

### Relationship between class of recall and recall characteristics

The class of recall exhibited substantial relationships with several recall characteristics **(**Table 7**)**. Notably, the year of the recall demonstrated a significant association with the class of recall. Class II recalls predominated across all years, comprising 56.5% in 2019, 54.5% in 2020, 52.6% in 2021, and 100% in 2022. The relationship between drug category and class of recall was evident, with Class II recalls being found more often with antibiotics (78.9%), anti-inflammatory drugs (100%), disinfectants (75.0%), antiparasitic drugs (100%), and vitamins (83.3%). Conversely, Class I recall was primarily found with spasmolytics (100%), antihypertensive drugs (62.5%), and diagnostic agents (60%).

**Table 7:** Relationship between class of recall and recall characteristics

		***N*** ** = 106**		**Chi** ^**2**^ ** or Fisher’s exact**
**Characteristics**	**Class I n (%)** 35 (33.0)	**Class II n (%)** 62 (58.5)	**Class III n (%)** 9 (8.5)	***p*** **-value**
**Year of recall**				**0.01**
2019	10 (43.5)	13 (56.5)	0 (0.0)	
2020	16 (29.1)	30 (54.5)	9 (16.4)	
2021	9 (47.4)	10 (52.6)	0 (0.0)	
2022	0 (0.0)	9 (100)	0 (0.0)	
**Drug category**				** < 0.001**
Antibiotic	0 (0.0)	30 (78.9)	8 (21.05)	
Anti-inflammatory	0 (0.0)	5 (100)	0 (0.0)	
Disinfectant	3 (25.0)	9 (75.0)	0 (0.0)	
Spasmolytic	17 (100)	1 (5.6)	0 (0.0)	
Antiparasitic	0 (0.0)	4 (100)	0 (0.0)	
Antihypertensive	5 (62.5)	3 (37.5)	0 (0.0)	
Vitamins	1 (16.7)	5 (83.3)	0 (0.0)	
Diagnostic agent	3 (60)	2 (40)	0 (0.0)	
Others	6 (60.0)	3 (30.0)	1 (10.0)	
**Safety issues**				** < 0.001**
Contamination	25 (89.3)	3 (10.7)	0 (0.0)	
Suspected poor quality	7 (35.0)	13 (65.0)	0 (0.0)	
Not labelled	0 (0.0)	9 (100)	0 (0.0)	
Clumping	0 (0.0)	8 (100)	0 (0.0)	
Color change	0 (0.0)	8 (100)	0 (0.0)	
Precipitation	0 (0.0)	7 (100)	0 (0.0)	
Self-opening capsules	0 (0.0)	0 (0.0)	8 (100)	
Others	3 (16.7)	14 (77.8)	1 (5.5)	
**Supplier**				
Non-governmental	28 (41.8)	30 (44.8)	9 (13.4)	**0.002**
Governmental	1 (7.1)	13 (92.9)	0 (0)	
Not specified	6 (24.0)	19 (76.0)	0 (0.0)	
**Reporter**				** < 0.001**
Rwanda FDA	0 (0.0)	25 (100)	0 (0.0)	
Rwanda Medical Supply Ltd	5 (23.8)	16 (76.2)	0 (0.0)	
Private pharmacies and firms	27 (69.2)	8 (20.5)	4 (10.3)	
Public/Government pharmacies	3 (27.3)	3 (27.3)	5 (45.4)	
Others	0 (0.0)	10 (100)	0 (0.0)	
**Recall level**				**0.01**
Retail	30 (29.7)	62 (61.4)	9 (8.9)	
Consumer	5 (100)	0 (0.0)	7 (0.0)	
**Top five manufacturing country**				** < 0.001**
India	6 (19.4)	25 (80.6)	0 (0.0)	
France	19 (100)	0 (0.0)	0 (0.0)	
China	3 (16.7)	7 (38.9)	8 (44.4)	
Kenya	0 (0.0)	13 (92.9)	1 (7.1)	
Russia	0 (0.0)	7 (100)	0 (0.0)	
*Others*	7 (41.2)	10 (58.8)	0 (0.0)	

Contamination issues featured prominently in Class I recalls (89.3%) compared to Class II recalls (10.7%). Suspected poor quality contributed to 35.0% of Class I recalls and 65.0% of Class II recalls. Mislabeling, clumping, color change and precipitation exclusively constituted Class II recalls. All instances of self-opening capsules were associated with Class III recalls.

Supplier demographics demonstrated association with the class of recall, where Class II recalls prevailed. Non-governmental entities supplied 41.8% of Class I recalled drugs, while governmental suppliers provided 7.1% of Class I recalls. Rwanda FDA exclusively reported Class II recalls, Rwanda Medical Supply reported both Class I (23.8%) and Class II (76.2%) recalls, private pharmacies reported recalls across all classes with Class I at 69.2%, Class II at 20.5%, and Class III at 10.3%. Government pharmacies (affiliated with public institutions) reported Class II recalls (45.5%), followed by an even distribution of Class I and II recalls (27.3% each).

Recall levels demonstrated relationship with the class of recall, with retail recalls predominantly belonging to Class II (61.4%), followed by Class I (29.7%) and Class III (8.9%). Conversely, all consumer-level recalls were associated with Class I.

The origin of the manufacturing countries showed an association with the class of recall. Recalled products from India were primarily Class II recalls (80.6%) or Class I recalls (19.4%). All recalled products from France were classified as Class I recalls. China's recalled products were dispersed across all classes, with Class III at 44.4%, Class II at 38.9%, and Class I at 16.7%. Kenya exclusively featured Class II recalls (92.9%) and a minor Class III representation (7.1%). Finally, recalled products from Russia were entirely categorized as Class II.

## Discussion

This study aimed to assess the characteristics of Rwanda FDA recall products and determine the association between the classes of recall and the recall characteristics. The study revealed that a large proportion of recalled product belonged to group A of the WHO, ATC coding system and antibiotics constituted 25.8% of the recalled products. Class I recall accounted for 33.0% of all recalls; contamination emerged as a leading cause, accounting for 26.4% of recalled products. An association was found between the class of recall and many recall characteristics including the year of recall, drug category, safety issues, reporter, and manufacturing country.

Using the ATC coding system, group A represented a large proportion of recalled products, followed by group J and group C. The ATC coding system classifies drugs according to the organ or system on which their active substances act, based on their chemical, therapeutic or pharmacological properties [12]. Group A stands for Alimentary tract and metabolism, group C stands for cardiovascular system, and group J stands for anti-infective for systemic use [12]. Group C can have consequences because they are used to manage cardiovascular diseases, and in certain cases, an ineffective treatment can impact the course of the disease.

Antibiotics constituted 38.5% of the recalled products, antiparasitic drugs accounted for 12.3%. Using defective or poor-quality antibiotics can lead to antibiotic misuse and overuse, which contribute to antimicrobial resistance [14–16]. This is an alarming finding in a low-income country like Rwanda where antibiotic misuse is of an issue and antibiotic resistance is emerging [15, 17–20]. Furthermore, in addition to the measures that are in place to protect antibiotics, the use of defective or poor-quality anti-parasitic agents would impede WHO control and response to eliminate soil transmitted helminths [21]. Therefore additional measures should be directed to protecting antiparasitic especially in countries where parasitic infestation, including malaria and helminthic infestation are still high [22–24].

In addition to the potential threat posed by defective anti-hypertensive, antibiotics, and antiparasitic drugs, it is crucial to recognize the significant impact of compromised medications on human lives. This adds another layer of complexity to the public health risks associated with sub-standard pharmaceuticals, as they directly jeopardize the lives of individuals who rely on these drugs for the management of critical health conditions. As we address the multi-faceted challenges of drug recalls, safeguarding public health takes on sensitive significance, with a particular emphasis on the wellbeing and safety of those vulnerable population at risk.

Disinfectants and antiseptics accounted for 11.3% of product recalls, a noteworthy proportion. This prominence is likely attributed to the temporal overlap of our study period with the era of COVID-19, during which the use of hand sanitizers and other disinfectants significantly increased compared with the periods preceding and following the pandemic. Nevertheless, despite the WHO declaration that COVID -19 no longer poses a Public Health Emergency of International Concern (PHEIC) [25], it remains imperative to sustain pharmacovigilance efforts concerning disinfectants and antiseptics. These substances serve various purposes, and substandard quality can potentially engender severe health consequences, particularly in the context of healthcare-associated or nosocomial infections and surgical site infections, in addition to harming the users [26, 27].

Contamination emerged as a leading cause of product recalls, with numerous instances identified to contain potential foreign materials. In a specific case involving ranitidine, a nitrosamine impurity classified as a probable human carcinogen was detected, prompting a voluntary recall by the manufacturer. The pattern of contamination as a significant factor in product recall is evident not only in Rwanda but also in the United States, Nepal and Zambia [4, 28, 29]. The prevalence of contamination as a leading cause emphasizes the critical importance of rigorous quality control protocols throughout the production and distribution chain.

Class I recall accounted for 33% of all recalls, with 75.4% of the recalls involving antihypertensive drugs and 60% related to diagnostic agents being classified as Class I. Class I recalls are typically associated with products that pose a significant risk of adverse health consequences or even death [2]. The prevalence of class I recalls among antihypertensive and diagnostic agents emphasise the importance of rigorous quality control, monitoring, and post-market surveillance among these drug categories.

All consumer level recalls were classified as Class I. Rwandan guidelines state three recall levels: wholesale, retail, and consumer. The wholesale level involves all entities engaged in the wholesale distribution of pharmaceutical products. The retails level encompasses public and private hospitals, retail pharmacies, clinical investigators, institution, conducting clinical investigations, healthcare practitioners, nursing homes and other retail outlets handling pharmaceuticals. Finally, the consumer level involves patients and other end users of pharmaceutical products [30].

The trends in recall incidences over time was notable, with 21.7% of drug recalled in 2019, and 51.9% recalled in 2020. This incidence declined to 17.9% in 2021 and to 8.5% in 2022. Given that the Rwanda FDA was established in 2018, the relatively low incidence of drug recall in 2019 may suggest an early stage of vigilance within the regulatory framework. The significant increase observed in 2020 could be attributed to heightened inspections in response to the changing circumstances of the COVID-19 pandemic coupled with the influx of COVID-19-related products into the market. The subsequent decline in drug recall in 2021 and 2022 may signify the stabilization of pharmaceutical products safety measures, improved quality control, adherence to safety regulations, and enhanced recall efficacy. However, further investigations are warranted to gain a deeper understanding of the factors underlying these shifts.

Non-governmental entities supplied most of the recalled products (63.2%), while governmental (affiliated with a public institution) entities supplied 13.2%. Non-governmental suppliers were more involved in Class I recalls, while governmental suppliers were more prevalent in Class II. This may call for a more thorough investigation of the procedures and quality control mechanism used by both governmental and non-governmental suppliers. This disparity may also reflect potential differences in oversight and regulation between these two supplier types.

India emerged as a predominant source of recalled products, accounting for 29.2% of the total recall but, many Indian products fell into Class II recalls (80.6%). India was also found to be the leading manufacturing country for the recalled product in Zambia, Nepal and Sri Lanka [28, 29, 31]. This highlights the need for heightened vigilance in quality control, within its pharmaceutical industry and underlines the importance of vigilance for products imported from India. This highlights the necessity of strengthening the regulatory system, primarily through the enhancement of inspections for good manufacturing practices, the improvement of registration procedures, and the enhancement of post-marketing surveillance for pharmaceutical products. To enhance pharmaceutical safety globally, collaborative efforts among countries, industry stakeholders, and regulatory bodies are important [32].

All recalled products from France were classified as class I recalls, and 100% of all spasmolytic drugs also fell into the class I recall category. This observation can be attributed to the noteworthy fact that multiple batches of Debridat, a spasmolytic medication, were subject to recalls because of contamination, and all these batches originated in France.

In the context of drug recall, various East African countries have distinct guidelines for regulating the process of drug recall, reflecting their unique legal and regulatory frameworks. In Rwanda, emphasis is placed on stakeholder responsibility, effective communication, and collaboration. The Rwanda FDA handles statutory (non-voluntary) recalls, while voluntary recalls are initiated by the market authorization holders (MAH) and manufacturer. The authority classifies recalls and sets timelines for initiation and completion, guiding the disposal of unfit products. The initiation timeline is set at 24 h for Class I recalls, 48 h for Class II, and 72 h for Class III. The completion timeline is within 72 h for class I, 48 h for Class II, and 30 days for Class III [30]. South Africa focuses on compliance with the Medicines and Related Substances Act and initiates recalls through certificate holders, importers, or South African Health Products Regulatory Authority (SAHPRA). All recalls involve consultation with SAHPRA, which agrees on recall strategy [33]. In Uganda, the National Drug Authority (NDA), requires importer to report, with NDA assessing risks and overseeing recalls [34]. Tanzania, under the Tanzania Medicines and Medical Devices Authority, regulates imports and exports and checks products quality at entry points [35]. In Kenya, recalls are initiated by the MAH and manufacturers, with Pharmacy and Poison Board (PPB) overseeing the process [36].

This study has limitations. First as a descriptive cross-sectional study including secondary analysis of drug recall data, data completeness was an issue. Second, this study could not identify the root causes of contamination, poor quality, and other safety issues that limit the ability to implement targeted preventive measures. Additionally, the study primarily focuses on Rwanda's pharmaceutical recall landscape; therefore, the results may not be generalizable. To address these limitations, future research should adopt a broader perspective, including comparisons with international recall practices, to offer valuable insights into global best practices and potential areas for improvement. Furthermore, exploring the influence of the regulatory framework on recall decisions and outcomes could provide a deeper understanding of the interplay between regulations, industry practices, and patient safety.

Based on the findings, it advisable for Rwanda FDA to prioritize actions that enhance the quality and safety of antibiotics in the market, given their high percentage in recalls and the potential impact on antimicrobial resistance. The Rwanda FDA should intensify its surveillance, monitoring, and post-market activities, particularly for anti-hypertensive drugs, given their predominance in Class I recalls, which carry significant health risk. Conducting a thorough investigation into the procedures and quality control mechanism of suppliers, especially governmental ones, is essential. Additionally, the Rwanda FDA should increase vigilance on imports from India emphasising rigorous inspections, improved registration procedures and enhanced post marketing surveillance. Finally, collecting more information on the registration status is crucial for enhancing regulatory effectiveness and ensuring pharmaceutical safety.

The Rwanda FDA and other regulatory agencies should closely collaborate with manufacturers, suppliers, and healthcare providers to implement proactive measures. Strengthening the regulatory system is key to reducing the frequency and impact of recalls, especially since many of the recalled products are vital drugs with significant implications for their use and the public health policies.

## Conclusion

In conclusion, this study provides a comprehensive overview of the characteristics of drug recalls in Rwanda. The insights gained contribute to a nuanced understanding of recall dynamics and provide evidence-based strategies to enhance drug quality, safety, efficacy, regulatory compliance, and patient welfare. These findings highlight the need for policymakers to prioritize the quality and safety of antibiotics, given their high percentage in recalls and their potential impact on antimicrobial resistance. Strengthening surveillance, monitoring, and post-market activities, especially for antihypertensive drugs, is imperative to mitigate the significant health risks associated with class I recall. Furthermore, investigating the procedures and quality control mechanisms of suppliers, particularly governmental entities; is essential for ensuring pharmaceutical safety. Overall, concerted efforts to strengthen regulatory systems and foster collaboration among stakeholders are essential to safeguard public health and promote pharmaceutical safety in Rwanda and beyond.

### Supplementary Information

Supplementary Materail 1.

## Data Availability

All data generated or analysed during this study are included in this published article and its supplementary information files.

## Abbreviations

ATC: Anatomical Therapeutic Chemical Classification
RMS: Rwanda Medical Supply
Rwanda FDA: Rwanda Food and Drug Authority
WHO: World Health Organization
