# Supplementary material for: A four-year assessment of the characteristics of Rwandan FDA drug recalls
Source: BMC Public Health. 2024 Jul 4;24:1784. doi: 10.1186/s12889-024-19245-8 (PMC11223409; doi:10.1186/s12889-024-19245-8)
Supplement: Supplementary file 1 — Supplementary Materail 1. [file 12889_2024_19245_MOESM1_ESM.docx]

# **TABLES AND FIGURES**

Table S1. Characteristics of drug recalls

| **Characteristics** | | **N (%)** |
| --- | --- | --- |
| **ATC Classification** | |  |
|  | A | 33 (31.1) |
|  | B | 1 (0.9) |
|  | C | 9 (8.5) |
|  | D | 1 (0.9) |
|  | G | 2 (1.9) |
|  | H | 1 (0.9) |
|  | J | 30 (28.3) |
|  | M | 1 (0.9) |
|  | N | 4 (3.8) |
|  | P | 6 (5.7) |
|  | V | 5 (4.7) |
|  | Other (%) | 13 (12.3) |
| **Drug category** | |  |
|  | Antibiotic | 38 (35.8.) |
|  | Anti-inflammatory | 5 (4.7) |
|  | Disinfectant | 12 (11.3) |
|  | Spasmolytic | 18 (16.9) |
|  | Antiparasitic | 4 (3.8.) |
|  | Antihypertensive | 8 (756) |
|  | Vitamins | 6 (5.7) |
|  | Diagnostic agent | 5 (4.7) |
|  | Others | 10 (9.4) |
| **Class of recall** | |  |
|  | I | 35 (33.0) |
|  | II | 62 (58.5) |
|  | III | 9 (8.5) |
| **Supplier** | |  |
|  | Non-governmental | 67 (63.2) |
|  | Governmental | 14 (13.2) |
|  | Not specified | 25 (23.6) |
| **Reporter** | |  |
|  | Rwanda FDA | 25 (23.6) |
|  | Rwanda Medical Supply Ltd | 21 (19.8) |
|  | Private pharmacies and firms | 39 (36.8) |
|  | Public/Government pharmacies | 11 (10.4) |
|  | Others | 10 (9.4) |
| **Recall level** | | 101 (95.3) |
|  | Retail | 5 (4.7) |
|  | Consumer |  |
| **Top five manufacturing country** | |  |
|  | India | 31 (29.2) |
|  | France | 19 (17.9) |
|  | China | 18 (17.0) |
|  | Kenya | 14 (13.2) |
|  | Russia | 7 (6.6) |
|  | *Others* | 17 (16.0) |

Figure S1: Recalls per year

Figure S2: Safety issues of recalls
